# Section 12(2) MHA approval process is fit for purpose

**DOI:** 10.1192/bjb.2020.40

**Published:** 2020-06

**Authors:** Masum Khwaja

**Affiliations:** Consultant Psychiatrist, Central and North West London NHS Foundation Trust, and Honorary Clinical Senior Lecturer, Imperial College School of Medicine, email: masum.khwaja@nhs.net

Rigby and McAlpine^1^ have confusingly conflated criticism of Section 12 Mental Health Act (s12 MHA) approval courses, attendance at which is only one of several statutory criteria for s12 approval, with criticism of the overall process of s12 approval. This letter addresses some of the limitations of the article, which in hindsight I'm sure Rigby and McAlpine would prefer to have entitled: ‘Are s12 approval courses fit for purpose?’

Before reading further, readers should understand the following.

• A s12-approved doctor is legally defined as ‘a medically qualified doctor who has been recognised under section 12(2) of the MHA as having specific expertise in the diagnosis and treatment of mental disorder’ and has had training in the application of the MHA.^2^
• The criteria for s12 approval are contained within the statutory instructions^3^ and represent the Government's requirements regarding the work experience, training and qualifications doctors need to possess before they can legally be considered to have the ‘specific expertise in the diagnosis and treatment of mental disorder’ required to be an s12-approved doctor.
• Regional s12 approval panels have robust governance structures and procedures in place, including audit, that ensure that only those doctors that meet the statutory criteria are approved.
• Doctors require only a basic working knowledge of the MHA to be involved in MHA assessments (MHAAs).
• Doctors do not need to be s12 approved to be involved in MHAAs. If they are not s12 approved then it is preferable that they have personally treated the patient in the past or have some previous knowledge of the patient's case.
• S12 approval courses are not courses on which doctors learn about the MHA, or how to conduct MHAAs, for the first time. The courses serve to reinforce and enhance attendees’ knowledge of the MHA and of the MHA Code of Practice. They offer valuable time for discussion among clinicians, with a solicitor present, with debate often focused on the intricacies of the MHA as opposed to the basics.
• Not all s12-approved doctors are actively involved in detaining patients under the MHA. Examples include medical members of the first-tier tribunals (mental health), Second Opinion Appointed Doctors (SOADs) and doctors who produce independent expert reports for court.

Aspects of Rigby and McAlpine's article that need highlighting include the following.

• Rigby and McAlpine imply that s12 doctors may not be ‘equipped with the knowledge and skills’, particularly knowledge of the MHA, to consider detention of patients under the MHA and that this may have contributed to a rise in detentions over the past decade. There is no evidence to support their view, which in any case has not considered that the decision to apply to detain a person under the MHA does not lie with s12 doctors but with, usually, an approved mental health professional (who makes the application).
• Rigby and McAlpine declare that a ‘lack of formative assessment [in relation to s12 course objectives] is particularly concerning considering that there is evidence to indicate that there are inadequacies in many psychiatrists’ understanding of the relevant [MHA] legislation’. Once again, this is an eye-catching assertion for which they offer no convincing evidence. The two papers they cite are more than 20 years old, from 1999 and 1997 respectively, and pre-date the introduction of routine s12 approval courses, which commenced around 2002.
• Rigby and McAlpine state that international applicants with MRCPsych may not be aware of the UK MHA as ‘the MRCPsych does not assess UK mental health law’. However, they neglect to mention that s12 legal instructions require that ‘if the applicant has completed all or a substantial part of their training outside England or Wales, that the applicant must provide evidence of ‘steps the applicant has taken to familiarise themselves with psychiatric practice and the organisation of psychiatric services in England or Wales, including the practical application of the 1983 Act’.^3^ Furthermore, the MRCPsych curriculum is different to the requirements for core training (e.g. Workplace Based Assessments (WBPA)), and the knowledge and skills pertinent to s12 approval are mostly gained outside preparation for the MRCPsych examination.
• In criticising references for s12 approval, Rigby and McAlpine fail to appreciate that references for s12 approval are not restricted to commenting on an applicant's ability to undertake an MHAA but also require referees to be able to comment on, for example, the applicant's report writing and attendance at legal hearings, or that referees are, as always, bound by General Medical Council guidance in that references must be honest, objective and include all information relevant to a colleagues’ competence, performance and conduct.^4^
• The response rate of their survey was only 21.7% (5/23), which is far too low for the results to be usefully interpreted regardless of triangulation.

In summary, Rigby and McAlpine offer no convincing evidence that attendees of s12 courses do not have a working knowledge of the MHA sufficient to undertake MHAAs, or that the introduction of an additional requirement for s12 approval, to pass a multiple choice question (MCQ) and clinical examination, essentially on the MHA and on conducting MHAAs, is required.

We would also argue that, other than theoretically, Rigby and McAlpine provide no evidence that s12 courses, as they are currently delivered, fail to fulfil their core objectives or require major revision, or that the s12 approval process is anything but fit for purpose.

It is always important to consider how training may be improved; in relation to s12 courses, as Rigby is aware, the London Approval Panel have suggested that benchmarking MCQ course material and content across s12 courses nationally might support the development of improved course material and potentially of a ‘s12 course MCQ self-assessment revision aid’ focused on the essential aspects of mental health law and the Code of Practice akin to mandatory training on the MHA recommended by the MHA Code of Practice and monitored by the Care Quality Commission.^5^

The development of continuing professional development material relevant to building or maintaining the skills and knowledge required to act as an s12-approved doctor, and support from employers to evidence experience pertinent to s12 approval and reapproval in annual appraisals, are areas that those interested in supporting doctors to improve practice in relation to their s12 work might also wish to focus on.
